# Supplementary figures and images for: Nova proteins direct synaptic integration of somatostatin interneurons through activity-dependent alternative splicing
Source: eLife. 2023 Jun 22;12:e86842. doi: 10.7554/eLife.86842 (PMC10287156; doi:10.7554/eLife.86842)

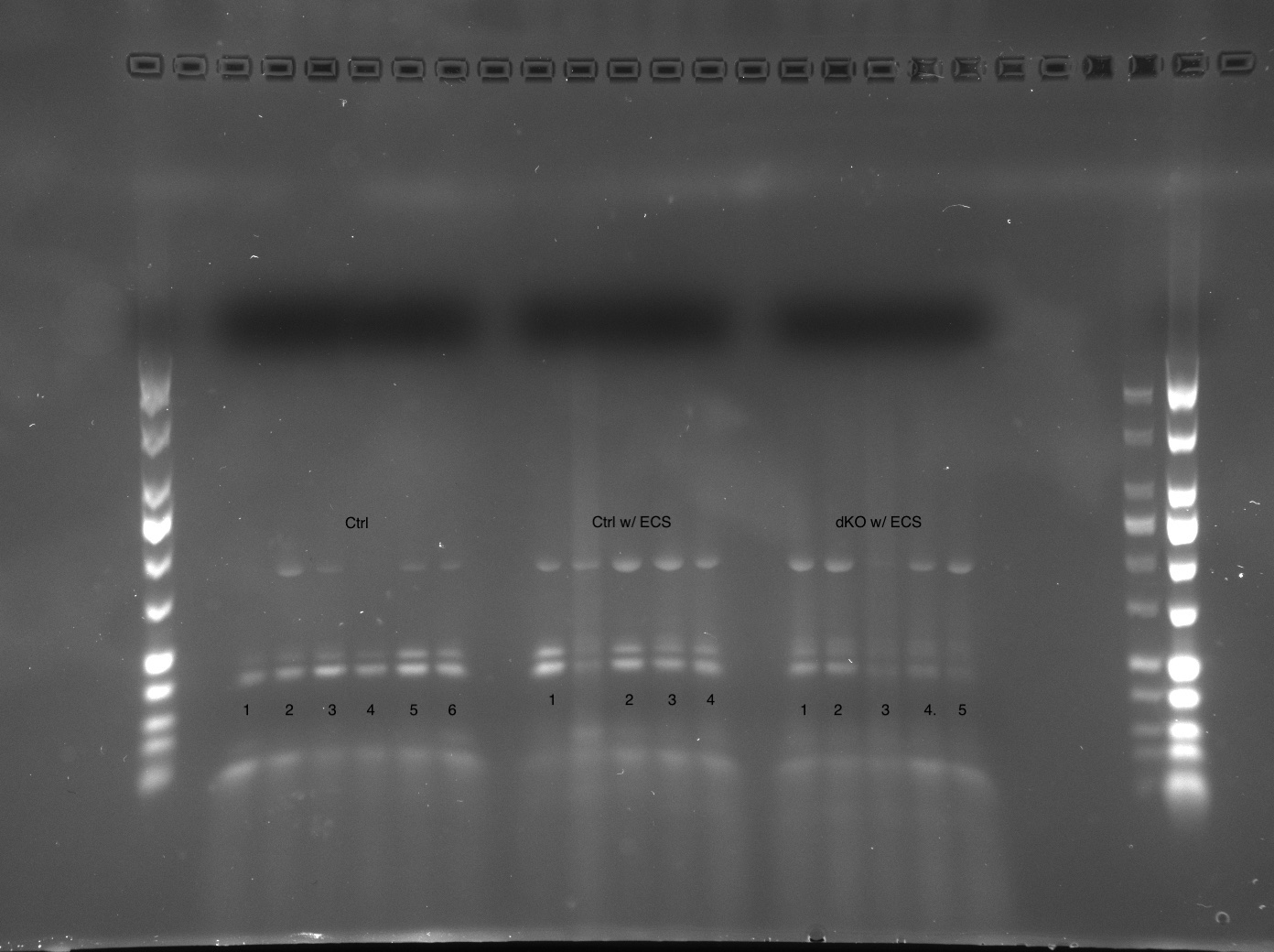

Supplement: Figure 6—source data 1. [file elife-86842-fig6-data1.zip › Figure 6G Source Data Labelled Gel.jpg]

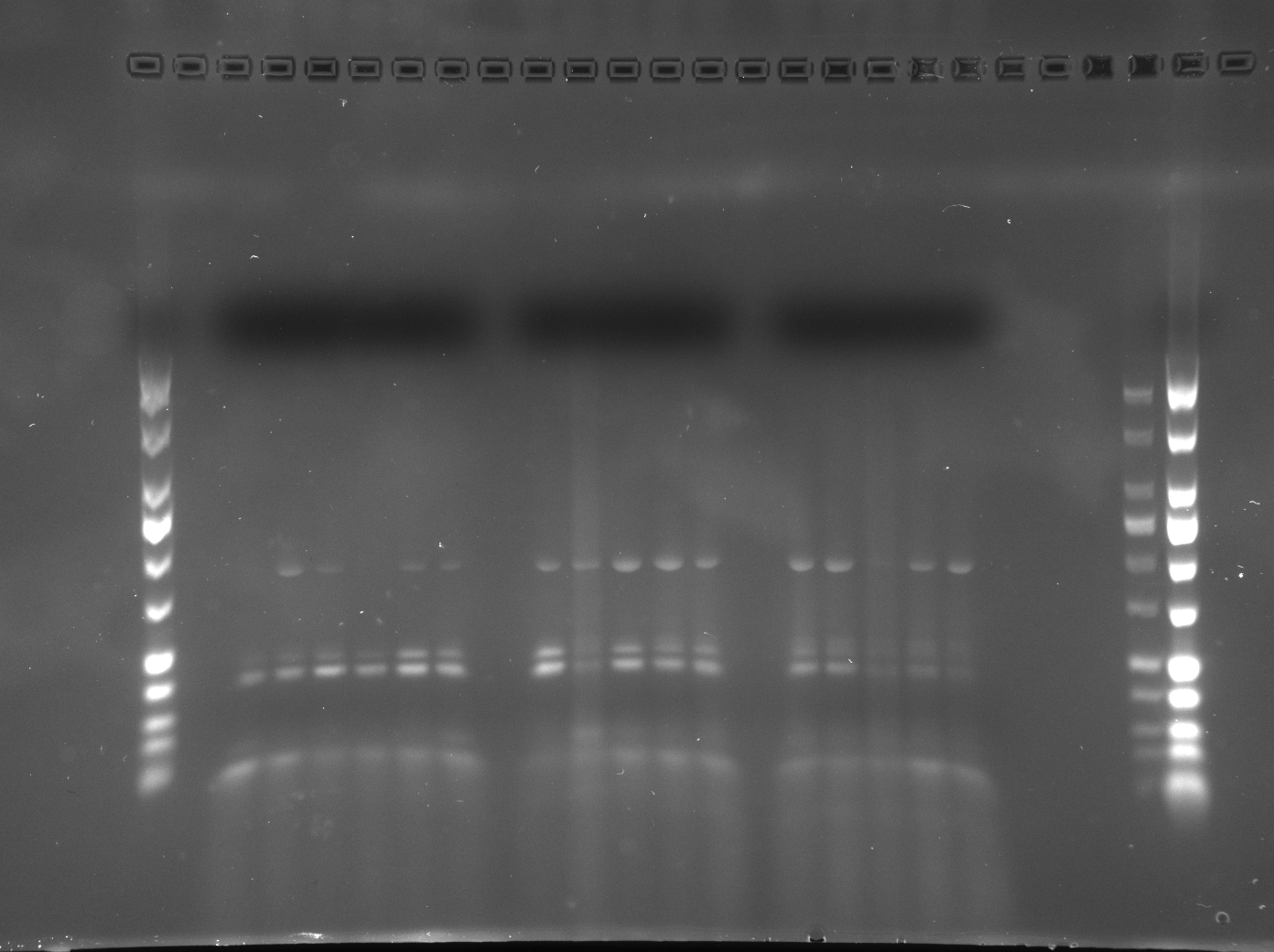

Supplement: Figure 6—source data 1. [file elife-86842-fig6-data1.zip › Figure 6 G Unedited Gel.tif]

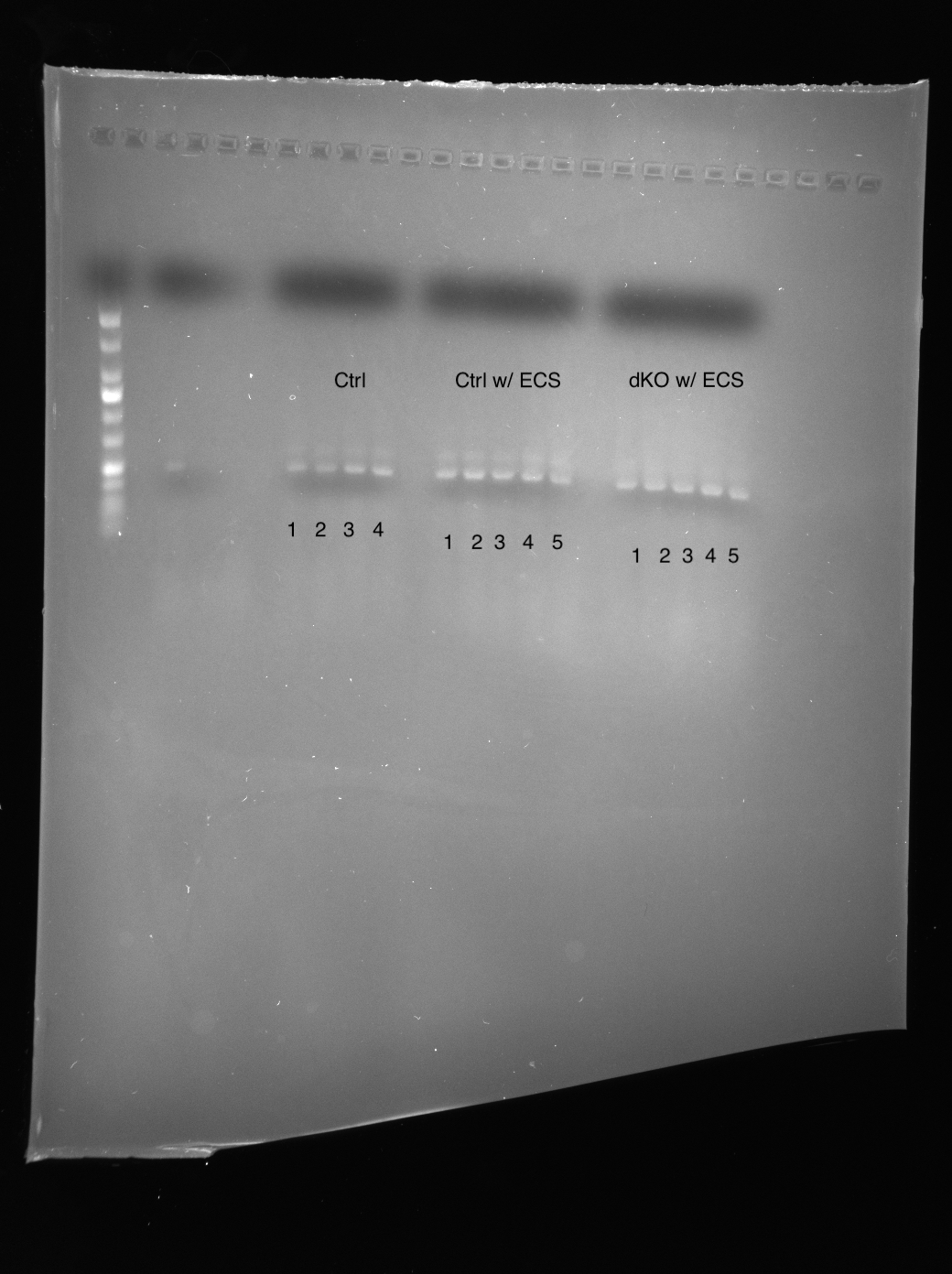

Supplement: Figure 6—figure supplement 1—source data 1. [file elife-86842-fig6-figsupp1-data1.zip › Supplementary figure S7 C Source Date Labelled Gel.tiff]

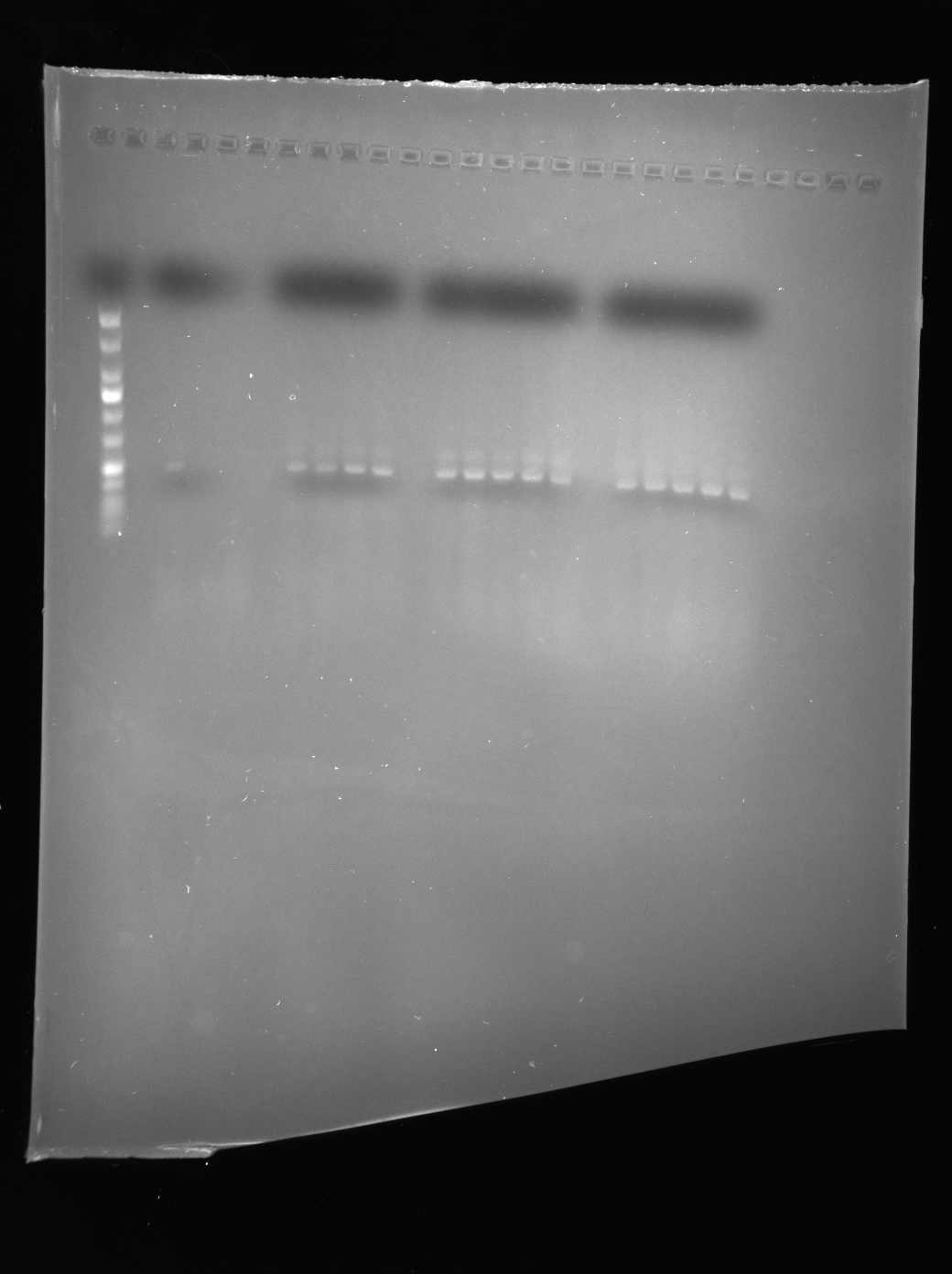

Supplement: Figure 6—figure supplement 1—source data 1. [file elife-86842-fig6-figsupp1-data1.zip › Supplementary figure S7 C Source Data Unedited Gel.tif]

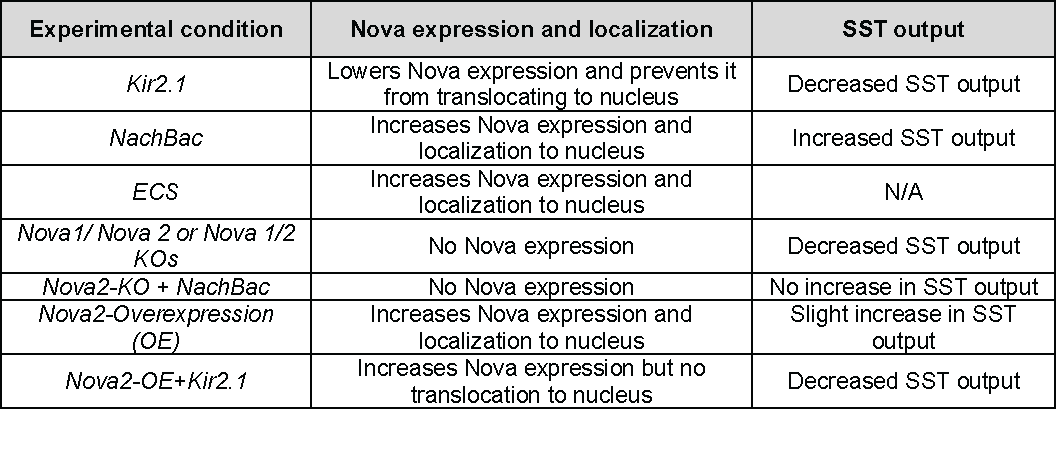


Figure 7-Source Data 1

Supplement: Figure 7—source data 1. [file elife-86842-fig7-data1.zip › Figure 7-Source Data 1.docx]
