# Supplementary material for: Nova proteins direct synaptic integration of somatostatin interneurons through activity-dependent alternative splicing
Source: eLife. 2023 Jun 22;12:e86842. doi: 10.7554/eLife.86842 (PMC10287156; doi:10.7554/eLife.86842)
Supplement: Supplementary file 1. [file elife-86842-supp1.docx]

|  | *Control* | *Nova1-cKO* | *Nova2-cKO* | *N1/N2 dKO* | *One-Way ANOVA*  *p*-*value* |
| --- | --- | --- | --- | --- | --- |
| Input resistance [M:] | 213±10 | 192 ± 15 | 250±18 | 250 ± 20 | 0.03 |
| Sag ratio | 1.05 ± 0.033 | 1.087 ± 0.037 | 1.077± 0.037 | 1.1±0.05 | 0.07 |
| Rheobase [pA] | 120± 25 | 83 ± 15 | 25±3 | 128±30 | 0.01 |
| Peak amplitude (First Spike) [mV] | 86±7 | 81±3 | 87±4 | 60±9 | 0.007 |
| AHP (First Spike) [mV] | -9.6 ± 4 | -9.7 ± 0.85 | -6±1 | -8.6 ± 1.1 | 0.41 |
| Halfwidth (First Spike) [ms] | 1.3 ± 0.3 | 1.6 ± 0.4 | 1.9±0.3 | 2.5 ± 0.24 | 0.002 |
| Threshold (First Spike) [mV] | -34.29 ± 3.08 | -42.24 ± 5.93 | -36.6±3.9 | -24.1 ± 4.5 | 0.0013 |

**Supplementary File 1: Intrinsic properties of *SST-Nova1*, *SST-Nova2* and *SST-Nova1/2-*dKO**
